# Respiratory muscle strength in stroke: a case-control study

**DOI:** 10.1590/1806-9282.20240061

**Published:** 2024-08-16

**Authors:** Abdurrahim Yildiz, Rustem Mustafaoglu, Ayse Nur Bardak

**Affiliations:** 1 Sakarya University of Applied Sciences, Department of Physiotherapy and Rehabilitation - Sakarya, Turkey.; 2 Istanbul University - Cerrahpasa, Department of Physiotherapy and Rehabilitation - İstanbul, Turkey.; 3 University of Health Sciences, Istanbul Physical Medicine and Rehabilitation Training and Research Hospital - İstanbul, Turkey.

**Keywords:** Stroke, Muscle strength, Rehabilitation

## Abstract

**AIM:**

The aim of the study was to determine the respiratory muscle strength of stroke patients and compare them with healthy individuals.

**METHOD:**

The study was conducted with 171 patients who had a stroke between 2017 and 2021 and 32 healthy controls. Respiratory muscle strength and inspiratory and expiratory mouth pressure (MIP and MEP) were measured using the portable MicroRPM device (Micro Medical, Basingstoke, UK).

**RESULTS:**

The stroke group exhibited significantly lower values in both MIP for men (p<0.001) and women (p=0.013) and maximal expiratory pressure for men (p<0.001) and women (p=0.042), compared with the healthy control group. Notably, there was a significant difference in the MIPmen (p=0.026) and MEPmen (p=0.026) values when comparing the reference values, which were calculated based on age and sex, with those of the healthy group. The baseline values calculated according to age for stroke patients were as follows: MIPmen 31.68%, MIPwomen 63.58%, MEPmen 22.54%, and MEPwomen 42.30%.

**CONCLUSION:**

This study highlights the significant respiratory muscle weakness experienced by stroke patients, with gender-specific differences. It highlights the importance of incorporating respiratory assessments and interventions into stroke rehabilitation protocols to improve the overall health and well-being of stroke patients.

## INTRODUCTION

Stroke is a significant contributor to long-term disability globally. Although the effects of stroke on motor and cognitive function have been extensively studied, its impact on respiratory muscle strength remains an area requiring further research^1^. Understanding the changes in respiratory muscle strength following a stroke is essential, as it directly influences a patient's capacity to breathe effectively and can have a substantial impact on their overall quality of life^2^.

The literature has revealed that stroke affects not only the upper and lower extremity muscles but also the muscles associated with the respiratory system^3,4^. Stroke survivors frequently exhibit characteristic alterations in their respiratory patterns, including reduced ventilation, diminished respiratory muscle strength, and decreased activity in the diaphragm on the affected side^5,6^. Furthermore, these alterations are linked to reduced respiratory function, deconditioning, decreased levels of physical activity, and an elevated risk of experiencing respiratory complications. Therefore, it is justifiable to prioritize interventions aimed at enhancing respiratory function in stroke patients to mitigate morbidity and mortality risks^7^. The assessment of respiratory muscle strength in individuals with stroke is of paramount importance because it can significantly decline compared with healthy individuals. Maximal inspiratory pressure (MIP) and maximal expiratory pressure (MEP) are common metrics employed to evaluate respiratory muscle strength. In reference studies on respiratory muscle strength, MIP and MEP values of 74.2 and 66.7%, respectively, were reported in stroke patients compared with healthy controls^3^, and another study reported lower values of 55.5% for MIP and 60.6% for MEP in stroke patients^8^.

The significance of respiratory muscle function within the context of stroke rehabilitation cannot be overstated. Impaired respiratory muscle strength can result in respiratory complications, reduced exercise tolerance, and a decline in functional independence. In summary, this study seeks to assess the respiratory muscle strength potential in stroke patients and provide a reference point for comparison with a healthy control group. As healthcare professionals continually refine stroke rehabilitation strategies, the findings of this study hold the potential to inform the development of targeted interventions aimed at enhancing respiratory muscle strength and, consequently, the overall quality of life for stroke survivors. By addressing the knowledge gap in this critical domain, we aimed to contribute to the advancement of stroke rehabilitation practices and foster a deeper comprehension of the multifaceted consequences of stroke on individuals’ lives. Therefore, the aim of this study was to determine the respiratory muscle strength of stroke patients and compare them with healthy individuals.

## METHODS

This study was conducted at a private neurological rehabilitation center in Istanbul Physical Therapy and Rehabilitation Hospital, as well as a public association catering to individuals with acquired stroke. Patient recruitment took place from April 11, 2017, to October 10, 2021.

### Participants

The stroke group consisted of 171 chronic stroke patients and the control group consisted of 32 age- and gender-matched healthy individuals. The inclusion criteria for stroke participants were as follows: (a) diagnosis of hemiplegia/hemiparesis; (b) being older than 18 years; (c) able to walk independently or with support (assistive devices, etc.); (d) able to understand instructions; and (e) willing to participate in the study. The inclusion criteria for control group participants were as follows: (a) being older than 18 years; (b) never smoked tobacco products (never smokers); (c) able to follow simple instructions; and (d) no pathology in visual ability and hearing. For all participants, the exclusion criteria were as follows: (a) not volunteering to participate in the study; (b) diagnosis of pulmonary disorder, severe cardiovascular disorders, and other neurological disorders; and (c) individuals receiving specialized cardiopulmonary training.

### Outcome measure

The respiratory muscles’ strength was evaluated by maximal inspiratory and expiratory pressures (MIP and MEP, respectively). The participants’ MIP and MEP were measured and recorded according to ATS/ERS criteria using a portable MicroRPM device (Micro Medical, Basingstoke, UK)^9^. The highest of at least three measurements that did not differ by more than 5 cm H_2_O was recorded for MIP and MEP. A percentage of the predicted values of MIP and MEP was specified as described by Black and Hyatt^10^.

### Statistical analysis

A descriptive analysis of the registered variables was conducted in this study. Demographic quantitative variables were reported as mean values along with their standard deviations (mean±SD), while qualitative variables were presented as absolute counts. For MIP and MEP, these values were expressed as percentages of the predictive values. The independent-sample t-tests were used. For comparisons, p<0.05 was considered statistically significant.

## RESULTS

The study encompassed a total of 171 participants in the stroke group and 32 healthy controls. Within the stroke group, 39.60% were male and 61.40% were female, while the healthy group consisted of 56.25% males and 43.75% females. Table 1 shows that there were no statistically significant differences between the groups in terms of gender, age, weight, height, and body mass index (p>0.05). In stroke patients, the proportion of affected sides was equally distributed between right and left. The average time since stroke onset was 388 days.

**Table 1:** Characteristics of the participants.

Variable	Stroke group (n=171)	Healthy group (n=32)	Differences between groups	
	Mean±SD n (%)	Mean±SD n (%)	Diff. means	p
Sex (male/female)	66 (39.60)/105 (61.40)	18 (56.25)/14 (43.75)		0.063
Age (years)	54.53±10.27	51.28±7.40	3.28	0.091
Height (cm)	167.05±8.14	165.50±8.44	1.55	0.326
Weight (kg)	76.69±12.52	72.66±16.25	4.03	0.113
BMI (kg/m^2^)	27.44±4.45	26.36±4.58	1.08	0.210
Time since stroke onset (days)	388.39±731.96			

BMI: body mass index; Diff. means; difference between the means of both groups.

Table 2 presents the results of MEP and MIP measurements for both the stroke group and the control group. The stroke group demonstrated significantly lower values in both MIP for men (MIPmen) (p<0.001) and women (MIPwomen) (p=0.013), and MEP for men (MEPmen) (p<0.001) and women (MEPwomen) (p=0.042), compared with the control group. Notably, there was a significant difference in the MIPmen and MEPmen values when comparing the reference values, which were calculated based on age and sex, with those of the control group. However, no significant difference was observed in the reference values for other parameters. The baseline values calculated according to age for stroke patients were as follows: MIPmen 31.68%, MIPwomen 63.58%, MEPmen 22.54%, and MEPwomen 42.30%.

**Table 2:** Comparison of respiratory muscle strength of groups.

Variable	Stroke group (n=171)	Healthy group (n=32)	Differences between groups		95%CI
	Mean±SD	Mean±SD	Diff. means	p	
MIPmen (cmH2O)	36.17±20.87	56.89±16.55	-20.72	<0.001*	-31.33 to −10.12
MIPmen (cmH2O) R.V.**	114.17±6.89	117.92±2.29	-3.75	0.026*	-34.85 to −10.23
MEPmen (cmH2O)	47.02±25.17	69.56±13.77	-22.54	<0.001*	-7.03 to −0.45
MEPmen (cmH2O) R.V.**	208.62±14.19	216.33±4.72	-7.71	0.026*	-14.49 to 0.93
MIPwomen (cmH2O)	47.52±23.59	64.43±22.39	-16.91	0.013*	-30.13 to −3.69
MIPwomen (cmH2O) R.V.**	74.74±5.70	77.12±5.08	-2.38	0.140	-28.94 to −0.54
MEPwomen (cmH2O)	59.05±25.14	73.79±25.62	-14.74	0.042*	-5.55 to 0.79
MEPwomen (cmH2O) R.V.**	139.59±5.92	142.06±5.28	-2.47	0.140	-5.77 to 0.82

CI: confidence interval

Diff. means

difference between the means of both groups

R.V.: reference value.

* Statistical significance.

** Reference values were calculated using Black and Hyatt predictive equations.

## DISCUSSION

Respiratory muscle strength in stroke patients is usually ignored in neurological rehabilitation training programs. We aimed to draw attention to this issue clinically and to obtain comprehensive data on the MIP and MEP assessment scores of patients in Türkiye. When compared with the healthy individuals and the reference values determined according to age, respiratory muscle strength was found to be significantly lower in stroke patients. According to the reference values, the results obtained in stroke patients were MIPmen 31.68%, MIPwomen 63.58%, MEPmen 22.54%, and MEPwomen 42.30%. The data clearly indicate that stroke patients exhibit lower values in MIP for both MIPmen and MIPwomen, as well as MEP for both MEPmen and MEPwomen compared with healthy individuals. This suggests that stroke has a significant impact on respiratory muscle strength and is consistent with the literature showing respiratory complications that can occur after stroke. It is also important to note that decreased respiratory muscle strength may lead to impaired lung function. A prior study reported mean values of MIP ranging from 17 to 57 in people after stroke, compared with approximately 100, and mean values of MEP ranging from 25 to 68, compared with approximately 120 cmH_2_O in healthy adults^11^. Comparison with the data of this study shows that we obtained results in a similar range.

According to the studies evaluating MIP and MEP values separately for men and women in the literature, Luvizutto et al. found 85.0±36.2 in males and 46.9±25.4 in females for MIP and 82.4±28.9 in males and 51.2±28.8 in females for MEP. When respiratory pressures were compared with the predicted value, a significant reduction in MIP was observed in men and women^12^. Ramos et al.^13^ determined the MIP and MEP responses as 71.85 and 62.28 for men and 57.75 and 49.50 for women. Compared with the values found in the literature, MIP was estimated as 105.41 and MEP as 114.79 for men and MIP as 80.57 and MEP as 78.46 for women^14^.

Comparing age-standardized reference values in the literature with data from patients with stroke, Lista Paz et al. found that both MEP and MIP values were significantly lower in the stroke group compared with the control group. In addition, MEP and MIP were <60% of the predicted values (51.56±20.83 and 51.41±20.85, respectively) in the stroke group^8^. Kubo et al. presented changes in respiratory muscle strength in three periods. The mean values of MIP and MEP data were 37.6±19.6, 44.3±24.8, 48.1±25.1 and 46.1±19.8, 55.8±26.5, 63.1±30.1, respectively^2^. Kim found MIP and MEP mean values of 31.17, 33.83 and 26.90, 29.03 in middle-aged and elderly stroke patients, respectively^14^. Jandt et al. found the mean values of MIP data as 36.71±21.22 and MEP data as 47.81±31.15^15^.

Our study also calculates reference values for MIP and MEP for stroke patients in relation to age. These reference values show that, on average, stroke patients have significantly lower MIP and MEP values than expected for their age group. By comparing baseline data from the control group with data from patients with stroke available in the literature, Ward et al. found that both MEP and MIP values were significantly lower in the stroke group compared with the control group^16^. An et al. measured MEP and MIP as 53.08±11.08, 52.50±10.47 and 39.67±5.91, 39.50±5.28 in the experimental and control groups^17^. Jo et al. measured MIP 20.41±3.72 in the intervention group and 18.53±2.47 in the control group and MEP 23.94±4.98 in the intervention group and 21.71±2.73 in the control group^18^. In the study conducted by Anjana in two groups, MIP and MEP values were measured as 45.81, 54.61 and 30.74, 30.33^19^.

According to the data of these studies in the literature, the number of studies on respiratory muscle strength in Turkey is limited. According to Boz et al., the MIP was 53.68±20.86 and the MEP was 61.44±22.46 in stroke patients^20^. According to the study conducted by Aydoğan Arslan et al., MIP and MEP values in the experimental group were 58.09±25.59 and 75.81±32.24, respectively, and 61.30±34.48 and 70.90±28.88, respectively, in the control group^21^. Comparison with the data of our study shows that MIP and MEP values are minimally lower. According to the reference values, it is observed that the sum of our female-male percentage data is similar. Due to the low number of people evaluated in both studies, we think that the levels and physical conditions of the patients may have caused the data to be higher.

Studies show that important changes at functional levels are found to be below 40%, which can lead to respiratory problems and recurrent hospitalizations^22^. Like these findings, other studies found a MIP lower than that predicted for individuals after stroke^14,22^.

## CONCLUSION

This evidence underscores the severe respiratory muscle weakness experienced by stroke patients, and gender-specific differences are also notable. Our results highlight the importance of incorporating respiratory assessments and interventions into stroke rehabilitation protocols to improve the overall health and well-being of stroke survivors. Further research is needed to examine more deeply the factors affecting respiratory muscle function in stroke patients and to develop targeted interventions in this vulnerable population.

## ETHICAL APPROVAL

Ethical approval for this study was given by the Istanbul Faculty of Medicine Clinical Research Ethics Committee on April 11, 2017, numbered 409. The study was conducted in accordance with the Declaration of Helsinki.
